# Numerical Analysis of Ca^2+^ Signaling in Rat Ventricular Myocytes with Realistic Transverse-Axial Tubular Geometry and Inhibited Sarcoplasmic Reticulum

**DOI:** 10.1371/journal.pcbi.1000972

**Published:** 2010-10-28

**Authors:** Yuhui Cheng, Zeyun Yu, Masahiko Hoshijima, Michael J. Holst, Andrew D. McCulloch, J. Andrew McCammon, Anushka P. Michailova

**Affiliations:** 1 Department of Bioengineering, University of California San Diego, La Jolla, California, United States of America; 2 Department of Computer Science, University of Wisconsin, Milwaukee, Wisconsin, United States of America; 3 Department of Medicine, University of California San Diego, La Jolla, California, United States of America; 4 Department of Mathematics, University of California San Diego, La Jolla, California, United States of America; 5 Department of Chemistry and Biochemistry, Department of Pharmacology, University of California San Diego, La Jolla, California, United States of America; 6 Howard Hughes Medical Institute, University of California San Diego, La Jolla, California, United States of America; University of Washington, United States of America

## Abstract

The t-tubules of mammalian ventricular myocytes are invaginations of the cell membrane that occur at each Z-line. These invaginations branch within the cell to form a complex network that allows rapid propagation of the electrical signal, and hence synchronous rise of intracellular calcium (Ca^2+^). To investigate how the t-tubule microanatomy and the distribution of membrane Ca^2+^ flux affect cardiac excitation-contraction coupling we developed a 3-D continuum model of Ca^2+^ signaling, buffering and diffusion in rat ventricular myocytes. The transverse-axial t-tubule geometry was derived from light microscopy structural data. To solve the nonlinear reaction-diffusion system we extended SMOL software tool (http://mccammon.ucsd.edu/smol/). The analysis suggests that the quantitative understanding of the Ca^2+^ signaling requires more accurate knowledge of the t-tubule ultra-structure and Ca^2+^ flux distribution along the sarcolemma. The results reveal the important role for mobile and stationary Ca^2+^ buffers, including the Ca^2+^ indicator dye. In agreement with experiment, in the presence of fluorescence dye and inhibited sarcoplasmic reticulum, the lack of detectible differences in the depolarization-evoked Ca^2+^ transients was found when the Ca^2+^ flux was heterogeneously distributed along the sarcolemma. In the absence of fluorescence dye, strongly non-uniform Ca^2+^ signals are predicted. Even at modest elevation of Ca^2+^, reached during Ca^2+^ influx, large and steep Ca^2+^ gradients are found in the narrow sub-sarcolemmal space. The model predicts that the branched t-tubule structure and changes in the normal Ca^2+^ flux density along the cell membrane support initiation and propagation of Ca^2+^ waves in rat myocytes.

## Author Summary

In cardiac muscle cells, calcium (Ca^2+^) is best known for its role in contraction activation. A remarkable amount of quantitative data on cardiac cell structure, ion-transporting protein distributions and intracellular Ca^2+^ dynamics has been accumulated. Various alterations in the protein distributions or cell ultra-structure are now recognized to be the primary mechanisms of cardiac dysfunction in a diverse range of common pathologies including cardiac arrhythmias and hypertrophy. Using a 3-D computational model, incorporating more realistic transverse-axial t-tubule geometry and considering geometric irregularities and inhomogeneities in the distribution of ion-transporting proteins, we analyze several important spatial and temporal features of Ca^2+^ signaling in rat ventricular myocytes. This study demonstrates that the computational models could serve as powerful tools for prediction and analyses of how the Ca^2+^ dynamics and cardiac excitation-contraction coupling are regulated under normal conditions or certain pathologies. The use of computational and mathematical approaches will help also to better understand aspects of cell functions that are not currently amenable to experimental investigation.

## Introduction

Ventricular cardiac muscle cells have deep invaginations of the extracellular space known as t-tubules [1]–[14]. In rodents, these invaginations branch within the cell to form a complex network that allows rapid propagation of the electrical signal (i.e. the action potential, AP) to the subcellular location (i.e. the sarcoplasmic reticulum, SR) where the intracellular Ca^2+^ required for the cell contraction is stored [14]. The release of Ca^2+^ from the SR depends on “trigger Ca^2+^” entering the cytosol from the extracellular space by activating sarcolemmal Ca^2+^ channels (L-type Ca^2+^ channels, LCC) and by Ca^2+^ entry via Na^+^/Ca^2+^ exchanger (NCX), [3], [9], [14], [15]. The trigger Ca^2+^ activates SR Ca^2+^ release channels (ryanodine receptors, RyRs) by the process of “Ca^2+^-induces Ca^2+^-release” (CICR) which amplifies the modest increase in intracellular Ca^2+^ concentration ([Ca^2+^]_i_) caused by the LCC and NCX influxes to provide sufficient Ca^2+^ for the proteins regulating muscle force (i.e. troponin C, TN) ), [14]. Thus, by working together, the microanatomy of t-tubules and SR permits spatially homogeneous and synchronized SR Ca^2+^ release and spatially uniform Ca^2+^ transients throughout the cell [5], [14], [16]. It has been also observed that the spatially uniform Ca^2+^ transients might be achieved if the SR Ca^2+^ release and uptake are abolished [5]. Yet, despite a wealth of information on ventricular cell function and structure, the mechanisms causing the synchrony of activation and the similarity of levels of [Ca^2+^]_i_ across the myocyte still remain unclear.

In cardiac muscle cells, several computational models have been introduced to investigate the Ca^2+^ signaling, buffering and diffusion [17]–[19] and Ca^2+^ wave initialization and propagation [12], [20]–[23]. All these studies, however, are conducted on simplified geometries (such as cylindrical or rectangular shapes) and it has been pointed out that a small geometric change (even in the case the change is uniformly applied) could greatly influence the suggested homogeneous Ca^2+^ distribution by initiating wave propagation in the computer simulation [20], [22]. Several laboratories, using common pool modeling approaches, have investigated also the effects of LCC and NCX distributions on global [Ca^2+^]_i_ transients in dyadic, sub-sarcolemmal and cytosol compartments [10], [24], [25]. Recently, to examine how the distribution of Ca^2+^ flux along the sarcolemma affects Ca^2+^-entry and Ca^2+^ diffusion and buffering, we developed a 3-D continuum model in rat ventricular cells [19]. An important limitation of this model is that a cylindrical t-tubule geometry was assumed while several studies have provided evidence that in rodent ventricular myocytes the realistic t-tubule geometry is quite complex (with large local variations in the diameter and transverse-axial anatomies), [9]–[12]. These experimental findings suggest that replacing our idealistic t-tubule model with a realistic geometry is needed. The use of idealistic shapes will change the diffusion distances and realistic Ca^2+^-transporting protein localizations in plane and depth directions and consequently the predicted [Ca^2+^]_i_ distributions.

In the present study, we sought to develop a morphologically correct geometric model of the t-tubule and to use this model for computational studies of the intracellular Ca^2+^ dynamics. We examined the Ca^2+^ signaling in rat ventricular myocytes that had been treated with ryanodine and thapsigargin to eliminate Ca^2+^ release and uptake by the SR. By using published electro-physiological data and laser-scanning confocal [Ca^2+^]_i_ measurements, we were able to analyze several important spatial and temporal features of the Ca^2+^ signals in these cells. In this context, our goal was at least three-fold. The first aim was to develop a mathematical model that would be in qualitative or quantitative agreement with published experimental measurements on Ca^2+^ influx, and Ca^2+^ buffering and diffusion in rat ventricular cells with SR function inhibited [5], [26]. Second, to use the model to investigate the importance of t-tubule ultra-structure and membrane Ca^2+^ flux distribution for the Ca^2+^ signals. The third task was to simulate the Ca^2+^ signals in the absence of fluorescent dye and to study the importance of the mobility of endogenous Ca^2+^ buffers (ATP and calmodulin) and altered extracellular Na^+^ ([Na^+^]_e_) for the Ca^2+^ signals. The analysis suggests that the quantitative understanding of the Ca^2+^ signaling requires more accurate knowledge of the t-tubule microanatomy and Ca^2+^ flux distribution along the sarcolemma. In agreement with experiment, with 100 µM Fluo-3, the lack of detectible differences in the depolarization-evoked Ca^2+^ transients was found when the Ca^2+^ flux was heterogeneously distributed along the sarcolemma. In the absence of Fluo-3, the predicted Ca^2+^ signals were strongly non-uniform. Even at modest elevation of Ca^2+^, reached during Ca^2+^ influx, large and steep Ca^2+^ gradients may develop in the narrow sub-sarcolemmal space. The model also predicts that branched t-tubule topology and changes in the normal Ca^2+^ flux density along the cell membrane support Ca^2+^ waves initiated at the sarcolemma. Preliminary results of this work have been presented to the Biophysical Society in abstract form [27].

## Materials and Methods

### 3-D imaging and geometric modeling of t-tubule microanatomy

**Figure 1:**
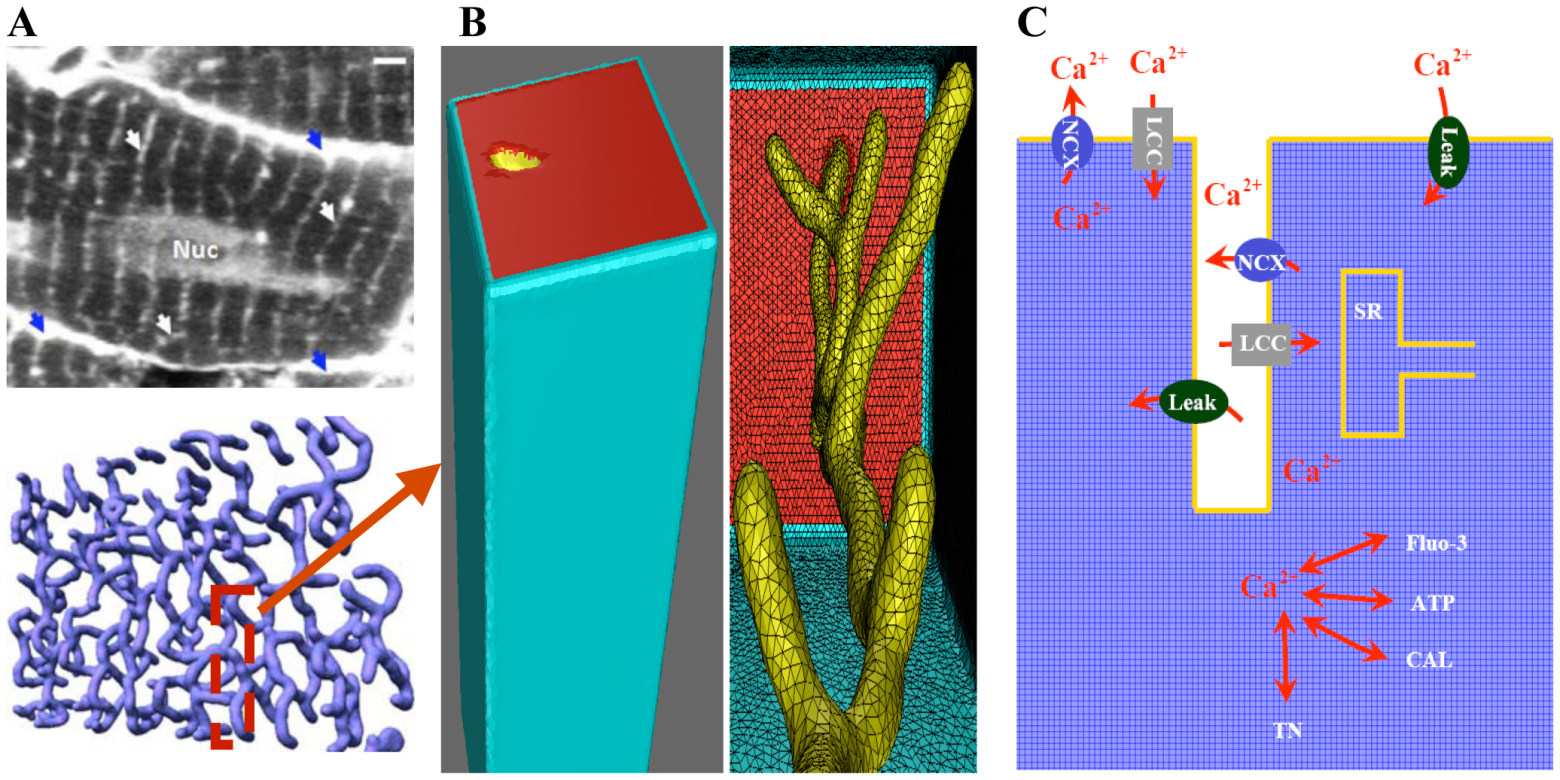
T-tubular network in mouse ventricular myoyctes, geometric model, and diagram illustrating Ca^2+^ dynamics. (A, *upper panel*) Cardiac sarcolemma including t-tubules is visualized using a 2-photon microscopy: external membrane (*blue arrows*); t-tubules (*white arrows*); nucleus (*Nuc*); bar 2 µm. (A, *lower panel*) Geometric model of the t-tubule sub-system extracted from 2-photon microscopy image. (B) Expanded view of single t-tubule geometry and its surrounding half-sarcomeres. (C) Schematic drawing of Ca^2+^ entrance and extrusion via the cell membrane and Ca^2+^ buffering and diffusion inside the myocyte with the SR activity inhibited.

Combining light microscopy (LM) and electron microscopy (EM) together with 3-D tomographic reconstruction, Hayashi *et al.* [6] investigated 3-D topologies of important sub-cellular organelles, including dyadic clefts and t-tubules, in mouse ventricular myocytes. In particular, the use of two-photon microscopy (T-PM) in their studies had provided data showing detailed spatial organization of t-tubules (see Fig. 1A upper panel) that was important for the development of our realistic model for computational studies of intracellular Ca^2+^ dynamics. The gap between imaging and simulation involves two major steps: (1) extracting features (boundary or skeleton) from imaging data; (2) constructing geometric models (represented by meshes) from the detected features. In addition, image pre-processing is usually necessary for better feature extraction, when the original image is noisy or the contrast between features and background is low. With 3-D T-PM images, Yu and collaborators developed a set of image processing and analysis tools and using the mesh generator called GAMer [28] they were able to generate high-fidelity and quality meshes for 3-D t-tubular systems in mice [29] (see Fig. 1A lower panel).

The extreme intricacy of the t-tubule system in mice (with transverse-axial anatomies and large local variations in t-tubule diameter) has been observed in rat ventricular myocytes as well [4], [11], [30]. Because high-fidelity geometric models representing the realistic t-tubule topology in rats are currently not available, in this study we used the geometric model in mice of Yu's *et al.* [29]. To investigate the Ca^2+^ signaling in rat ventricular myocytes, we considered a small compartment containing a single t-tubule and its surrounding half-sarcomeres for two reasons: (a) the entire t-tubular system in a ventricular myocyte forms a roughly periodic pattern corresponding to individual sarcomere; and (b) a small model contains much fewer number of mesh nodes that would render numerical simulation significantly faster and more feasible in ordinary computers. The surrounding half-sarcomeres were modeled as a rectangular-shaped box of 2 µm×2 µm in the plane of external sarcolemma and 5.96 µm in depth (Fig. 1B left panel and Table 1). As Yu's t-tubule model did not include the realistic cell surface, one of the box faces (the top red surface in Fig. 1B) was assumed to be the external cell membrane. The t-tubule inside this compartment was extracted from a sub-volume of the T-PM imaging data corresponding to the region indicated in Fig. 1A lower panel. To make it easier to handle boundary conditions in numerical analysis, we have “closed” the end of each branch, yielding a tree-like t-tubule model (see the yellow mesh in Fig 1B). These added “caps” were treated with the same boundary conditions as for the rest of the t-tubular surface. This simplified treatment clearly could introduce some errors because these “caps” are artificial and no Ca^2+^-transporting protein should reside there. However, the errors should be negligible as the area of these “caps” is very small, compared to the rest of t-tubular surface. The t-tubule diameter varied from 0.19 µm to 0.469 µm and the t-tubule depth was 5.645 µm. The volume of the model compartment was estimated to be ∼23.31 µm^3^. The compartment membrane area was ∼9.00 µm^2^ where the percentage of cell membrane within t-tubule was 64% (∼5.75 µm^2^) and within the external membrane 36% (∼3.25 µm^2^), [10], [11], [31], [32]. The accessible volume for Ca^2+^ was estimated to be ∼35–37% of the total cytosolic volume (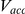) ∼12.9–13.6 pL in adult rat ventricular myocytes [33], [34]. The sub-cellular aqueous volume of 35–37% assumes that: (1) myofilaments occupy 47–48% of the cell volume, mitochondria 34–36%, nucleus 0–2%, t-tubule system 0–1.2%, and SR lumen 3.5%; (2) 50% of the myofilament space is accessible for Ca^2+^ (i.e. contains water); (3) mitochondria and nuclei are not rapidly accessible for Ca^2+^; (4) the SR lumen is not accessible to Ca^2+^ in the presence of ryanodine and thapsigargin [18], [33].

**Table 1:** Physical constants and cell geometry parameters.

Symbol	Definition	Value	Ref.
F	Faraday constant	96.5 C mmol^−1^	Physical constant
T	Temperature	295 K	Physical constant
R	Universal gas constant	8.314 J mol^−1^ K^−1^	Physical constant
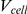	Cell volume	36.8 pL	[33]
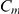	Cell capacitance	324 pF	[33]
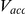	Accessible volume for Ca^2+^	12.9–13.6 pL	Estimated
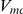	Compartment volume	23.31 µm^3^	Estimated
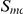	Compartment surface	9.00 µm^2^	Estimated
***T-tubule geometry***			
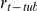	T-tubule radius	0.19–0.469 µm	[6]
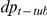	T-tubule depth	5.645 µm	[6]
***Rectangular-shaped box geometry***			
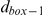	Cell surface direction	2µm	[12]
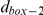	Cell surface direction	2µm	[12]
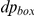	Depth	5.96µm	[6]

### Distribution of Ca^2+^-transporting protein complexes along the cell membrane

Recent immunohistochemical studies have demonstrated that marked variations in the distribution of Ca^2+^-transporting protein complexes (L-type Ca^2+^ channel, Na^+^/Ca^2+^ exchanger) along the cell membrane probably exist [2], [10], [12], [15], [35]–[43]. The analysis suggests that most of the L-type Ca^2+^ channels are concentrated in the t-tubules (from 3 to 9 times more in the t-tubule membrane than on the external sarcolemma) [2], [10], [12], [38], [39] and that the concentration of LCC along the t-tubule probably increases toward the center of the cell [36], [40].

Studies on the distribution of the main Ca^2+^ efflux pathway, the Na^+^/Ca^2+^ exchanger, are more controversial. All studies but one [41] have reported NCX to localize both to the external and t-tubule membrane, but most studies suggest that the NCX is 1.7 to 3.5 times more concentrated in the t-tubule membrane [15], [34], [36], [42], [43]. However, Kieval *et al.* data [43] indicate the NCX is more evenly distributed. In summary, the observed differences in the spatial distribution and molecular architecture of Ca^2+^ microdomains suggest that significant differences in the excitation-contraction coupling between the cell surface and cell interior may be exist. However how the localization of Ca^2+^- transporting protein complexes along the sarcolemma regulates the intracellular Ca^2+^ signaling still remains uncertain.

### Reaction-diffusion equations

In the current model, the effects of four exogenous and endogenous Ca^2+^ buffers (Fluo-3, ATP, calmodulin, troponin C) were considered (Fig. 1C). The endogenous stationary buffer troponin C (TN) was distributed uniformly throughout the cytosol but not on the cell membrane and in the sub-sarcolemmal space (∼40–50 nm in depth). The free Ca^2+^ and mobile buffers (Fluo-3, ATP, calmodulin) diffuse and react throughout the cytoplasm. The cell membrane and sarcomere box faces are subject to reflective boundary conditions. The nonlinear reaction-diffusion equations describing Ca^2+^ and buffers dynamics inside the model cell are:
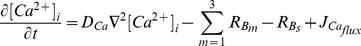
(1)

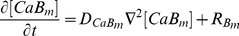
(2)

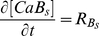
(3)

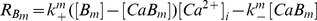
(4)

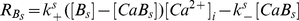
(5)where: [B_m_] represents the concentration of mobile buffer Fluo-3, ATP or calmodulin; [B_s_] is the concentration stationary buffer troponin C.

The diffusion coefficients for Ca^2+^, CaATP, CaCal and CaFluo as well as the total buffer concentrations and buffer rate constants used in the model are shown in Table 2. In the model we also assume: (1) isotropic diffusion for Ca^2+^ and all mobile buffers [12]; (2) Ca^2+^ binds to Fluo-3, calmodulin, ATP, and TN without cooperativity; (3) the initial total concentrations of the mobile buffers are spatially uniform; (4) the diffusion coefficients of Fluo-3, ATP or calmodulin with bound Ca^2+^ are equal to the diffusion coefficients of free Fluo-3, ATP or calmodulin.

**Table 2:** Calcium and buffer reaction-diffusion parameters.

Symbol	Definition	Value	Ref.
***Ca^2+^ and buffer concentrations***			
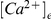	Extracellular Ca^2+^ concentration	1000 µM	[33]
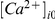	Resting Ca^2+^ concentration	0.1 µM	[33]
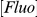	Total Fluo-3 concentration	100 µM	[18]
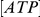	Total free ATP concentration	260 µM	[18]
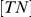	Total troponin concentration	70 µM	[18]
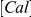	Total calmodulin concentration	24 µM	[18]
***Diffusion coefficients (at 22°C)***			
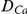	Diffusion coefficient for Ca^2+^	0.39 µm^2^ ms^−1^	[18]
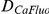	Diffusion coefficient for CaFluo	0.1 µm^2^ ms^−1^	[18]
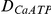	Diffusion coefficient for CaATP	0.168 µm^2^ ms^−1^	[18]
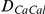	Diffusion coefficient for CaCal	0.025 µm^2^ ms^−1^	[18]
***Rate coefficients and dissociation constants (at 22°C)***			
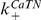	Ca^2+^ on-rate constant for TN	0.04 µM^−1^ ms^−1^	[19]
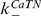	Ca^2+^ off-rate constant for TN	0.04 ms^−1^	[19]
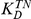	Ca^2+^ dissociation constant for TN	1 µM	[19]
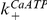	Ca^2+^ on-rate constant for CaATP	0.225 µM^−1^ ms^−1^	[18]
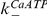	Ca^2+^ off-rate constant for CaATP	45 ms^−1^	[18]
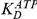	Ca^2+^ dissociation constant for ATP	200 µM	[18]
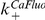	Ca^2+^ on-rate constant for CaFluo	0.23 µM^−1^ ms^−1^	[18]
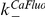	Ca^2+^ off-rate constant for CaFluo	0.17 ms^−1^	[18]
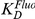	Ca^2+^ dissociation constant for Fluo	0.739 µM	[18]
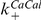	Ca^2+^ on-rate constant for Cal	0.125 µM^−1^ ms^−1^	[18]
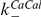	Ca^2+^ off-rate constant for Cal	0.2975 ms^−1^	[18]
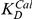	Ca^2+^ dissociation constant for Cal	2.38 µM	[18]

The total Ca^2+^ flux (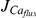) throughout the t-tubule and external membrane is:
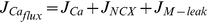
(6)where: 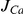 - total LCC Ca^2+^ influx; 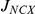
- total NCX Ca^2+^ flux; 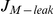
- total membrane Ca^2+^ leak.

To describe L-type Ca^2+^ current, Na^+^/Ca^2+^ exchanger, Ca^2+^ leak current densities we used the following expressions:
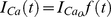
(7)

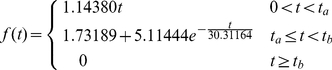
(8)

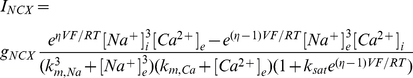
(9)

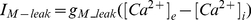
(10)


Flux parameter values were estimated or taken from the literature (see Table 3). In this study, the Ca^2+^ leak is not actually a particular “leak protein”. The Ca^2+^ leak was included and adjusted so that at rest Ca^2+^ influx via Ca^2+^ leak to match Ca^2+^ efflux via NCX thus no net movement across the cell membrane to occur.

**Table 3:** Membrane calcium fluxes parameters.

Symbol	Definition	Value	Ref.
***L-type Ca^2+^ current***			
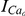	Constant	1	[18]
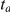	Constant	4 ms	[26]
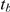	Constant	70 ms	[26]
***Na^+^/Ca^2+^ exchange current***			
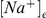	Extracellular Na^+^ concentration	140 mM	[33]
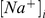	Resting Na^+^ concentration	10 mM	[33]
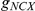	Pump rate of NCX	38.5 µM ms^−1^	[70]
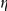	Voltage dependence of NCX control	0.35	[70]
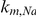	Na^+^ half saturation of NCX	87.5 mM	[70]
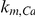	Ca^2+^ half saturation of NCX	1380 µM	[70]
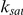	Low potential saturation factor of NCX	0.1	[70]
***Membrane Ca^2+^ leak***			
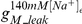	Conductance	3.4e-6µM mV^−1^ms^−1^	Estimated
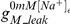	Conductance	−6.8e-6µM mV^−1^ms^−1^	Estimated

In the model, each current density (I_i_) was converted to Ca^2+^ flux (J_i_) by using the experimentally suggested surface to volume ratio (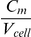
∼8.8 pF/pL) in adult rat ventricular myocytes [32], [33]:
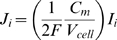
(11)Then, the total compartment Ca^2+^ flux (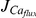) was computed by multiplying each total J_i_ with the model cell volume (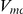), and distributing 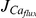 to the external and t-tubule membrane according to the prescribed Ca^2+^-handling protein concentration ratio.

The voltage-clamp protocol (holding potential −50mV, electric pulse of 10mV for 70ms) and whole-cell L-type Ca^2+^ current were derived from Zahradnikova *et al.* data with the blocked SR activity [26]. In this study, each simulation started with a basal cytosolic Ca^2+^ of 100 nM, basal cytosolic Na^+^ of 10 mM and buffers in equilibrium. The extracellular Ca^2+^ concentration (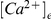) was 1 mM and remained constant. Unless specified otherwise in the Figure legends or in the text, the extracellular Na^+^ concentration (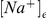) was 140 mM and 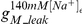 3.4^−6^ µM mV^−1^ ms^−1^.

### Numerical algorithms and software

In finite element methods, a complex domain needs to be discretized into a number of small elements (such as triangles or tetrahedra). This process is usually referred to as mesh generation [28], [44]. Although different types of meshes may be generated depending on the numerical solvers to be employed, we restrict ourselves to triangular (surface) and tetrahedral (volumetric) mesh generation as commonly used in biomedical simulation. In the present simulation, the number of finite element nodes and tetrahedral elements are 50,262 and 221,076, respectively.

The nonlinear reaction diffusion system was solved by a finite difference method in time and finite element method in space using our SMOL software tool (Smoluchowski Solver, http://mccammon.ucsd.edu/smol/) with the time step of 4 ms. It takes around 20 minutes to run 400 ms snapshots with a single Intel Xeon X5355 processor. The SMOL program utilizes libraries from the finite element tool kit (FETK), which previously has been used in several molecular level studies [45]–[49]. One bottleneck for dynamic 3-D simulation of nonlinear reaction diffusion system is the computing complexity involved in solving the problem. Here we successfully extended SMOL to solve multiple coupled partial differential equations with nonlinear ordinary equations. Multiple tests demonstrate that our SMOL program is quite robust and flexible for various boundary and initial conditions. The simulation results were visualized using GMV mesh viewers [50]. Post-processing and data analyses were implemented by customized Python, MATLAB 2008b (The MathWorks, Natick, MA) scripts and Xmgrace software [51]. A version control system, subversion, was used to monitor the development of software [52].

## Results

### Numerical simulation of experimental recordings of Ca^2+^ influx and Ca^2+^ concentration changes in rat ventricular myocytes

**Figure 2:**
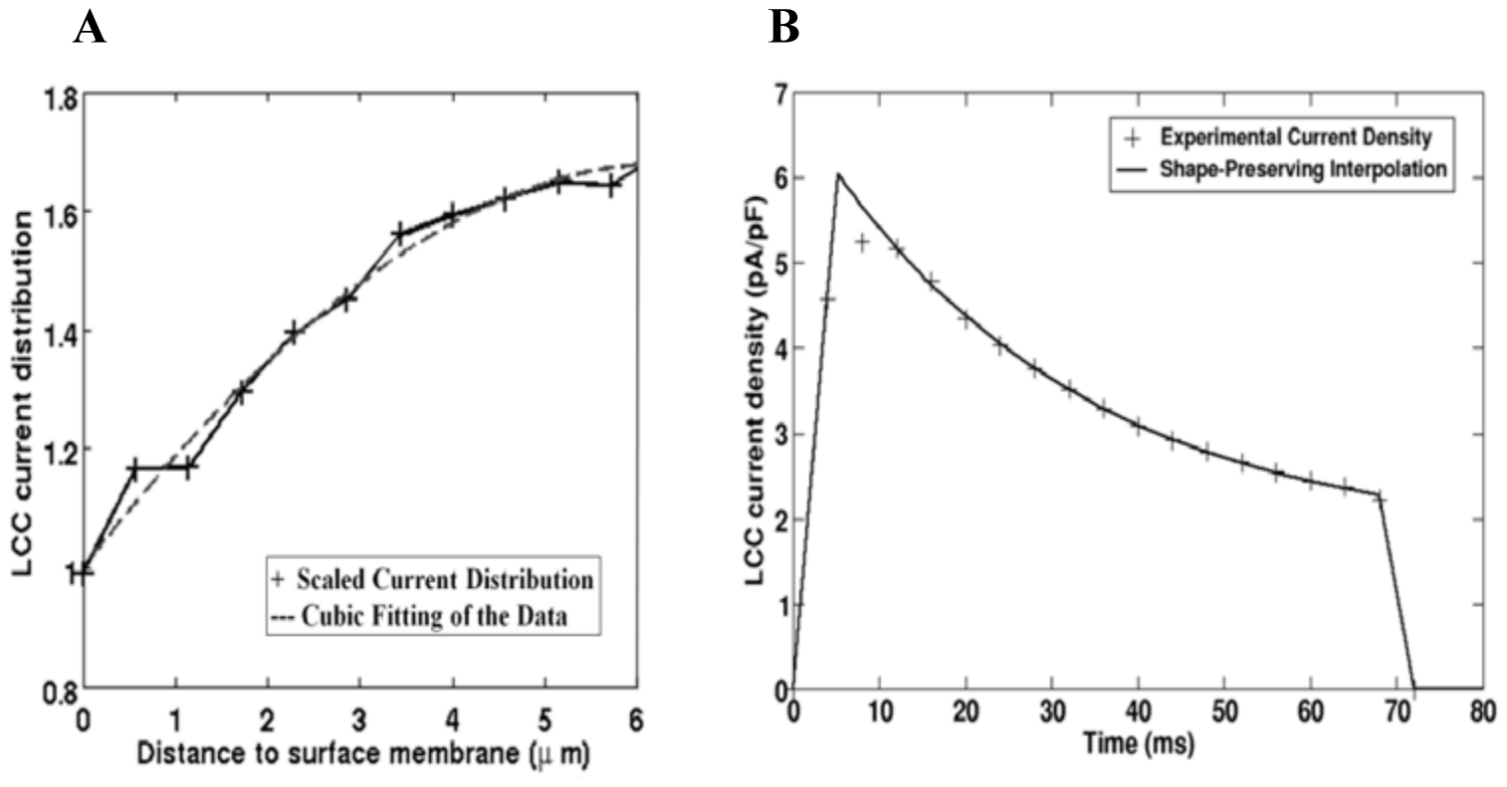
L-type Ca^2+^ current. (A) The distribution of L-type Ca^2+^ current is computed (*dashed line*) by multiplying the experimentally measured cluster density and fluorescent intensity plots (*solid line*), [36]. (B) The L-type Ca^2+^ current density is fitted and plotted (*solid line*) using shape preserving function of the data reported in rats with SR blocked (*doted line*), [26].

In agreement with the reported experimental data [2], [10], [12], [36], [38]–[40], the spatial patterns of [Ca^2+^]_i_ were calculated assuming LCC current density: (1) heterogeneously distributed along the cell surface; (2) six times higher and uniform in the t-tubule membrane; or (3) homogeneously distributed along the sacrolemma. In cases (1–2) the NCX flux density was assumed three times higher in the t-tubule and in case (3) NCX was evenly distributed along the sarcolemma. In this study, Ca^2+^ leak density was homogeneously distributed along the cell membrane with respect to all distribution choices of LCC and NCX. In case (1), the 3-D distribution of LCC current was computed by combining the cluster density and fluorescent intensity plots, see Fig. 2A. The data were then scaled and fitted by a cubic polynomial:
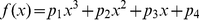
(12)where: *x* is the distance from the external cell surface.

The parameter values of the polynomial (p_j_, j = 1–4) are shown in Table 4. This polynomial was further scaled by a single factor C (see Table 4) such that the total Ca^2+^ flux along the t-tubule membrane remained unchanged by redistributing the Ca^2+^ fluxes. To fit the whole-cell LCC current density to the reported data in rat myocytes with SR release inhibited [26], we used a shape preserving function, (see Eq. 8 and Fig. 2B).

**Table 4:** Parameter values of the polynomial for L-type Ca^2+^ current distribution along the t-tubule.

Symbol	C	p_1_	p_2_	p_3_	p_4_
***L-type Ca^2+^ current***	0.4515	−4.1379e-4	−1.1722e-2	1.978e-1	1.0033

**Figure 3:**
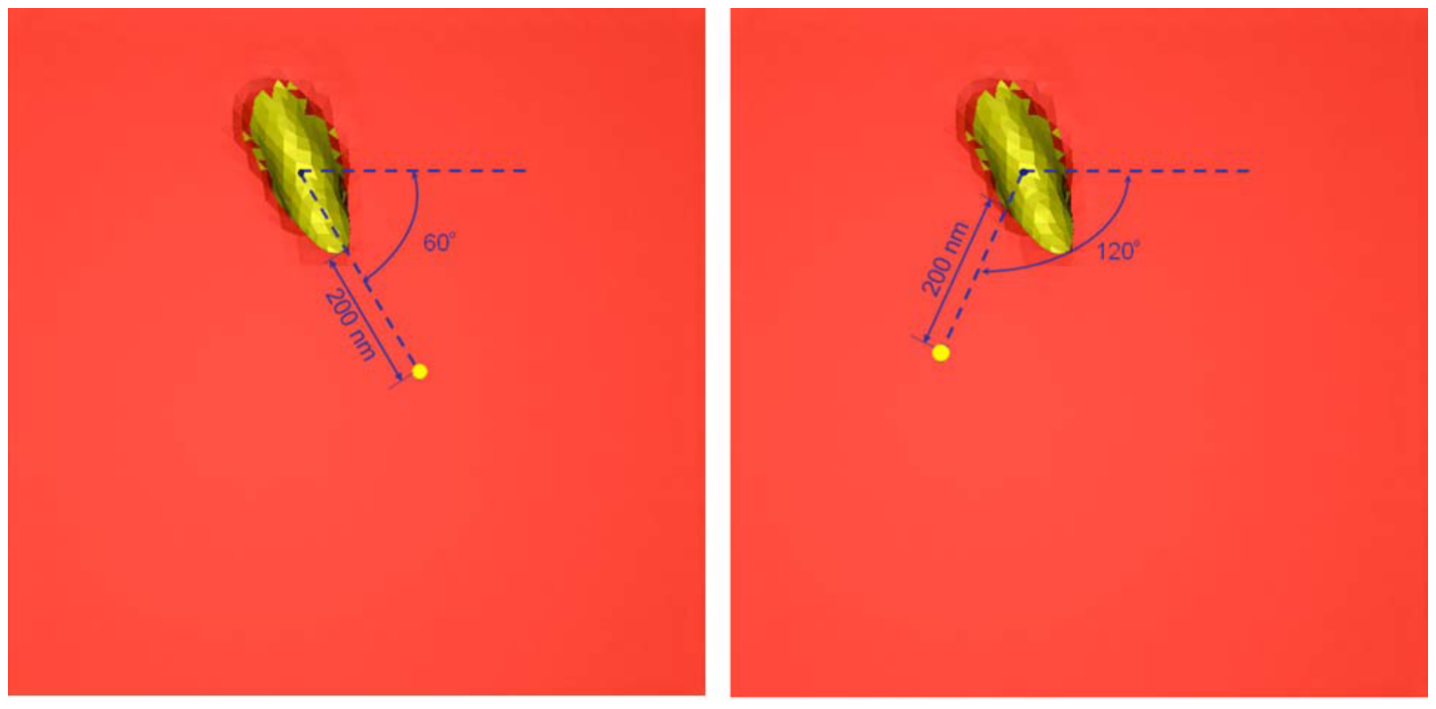
Line-scan position. Diagrams illustrating external membrane, t-tubule mouth, and positions of scanning line (*yellow spots*) where the changes in local [Ca^2+^]_i_ have been visualized and examined.

Consistent with the Cheng *et al.* experiment [5], where the fluorescence signal was recorded along the single scan-lane starting and ending outside the cell and crossing the center of the cell, the model t-tubule was chosen to cross the cell center and the scanned line was located at 200nm away from the t-tubule membrane (see Fig. 1A and Fig. 3). To gain more detailed insights of how the predicted Ca^2+^ signals are regulated within this geometrically irregular micro-domain we examined [Ca^2+^]_i_ at two different line-scan positions: 200 nm, angle 120°; 200 nm, angle 60° (see Fig. 3).

**Figure 4:**
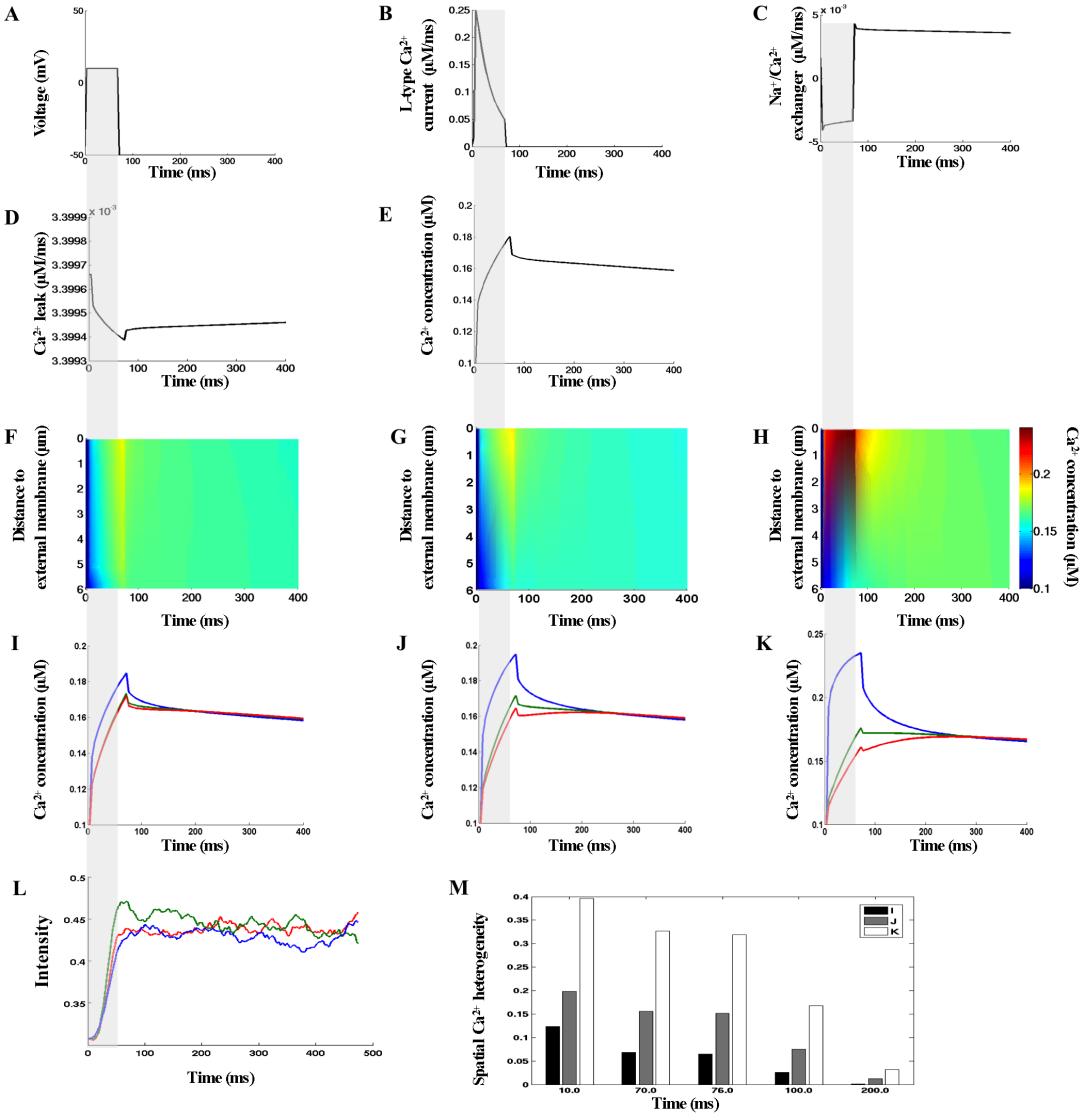
Calcium signals arising from the ionic fluxes via the external and t-tubule membrane in the presence of 100 µM Fluo-3. (A–B) The voltage-clamp protocol and whole-cell L-type Ca^2+^ current. (C–E) Predicted global Na^+^/Ca^2+^ and Ca^2+^ leak currents and global Ca^2+^ transient when no detectible differences in [Ca^2+^]_i_ are found (see panel F). (F–H) Calcium concentrations visualized as line-scan images in transverse cell direction. (I–K) Local Ca^2+^ transients taken at three featured spots along the scanning line of interest: 0.17 µm – *blue lines*; 3.09 µm – *green lines*; 5.45 µm – *red lines*. In (F) and (I) the L-type Ca^2+^ current density followed heterogeneous distribution along the length of t-tubule as shown in Fig. 2A. In (G) and (J) the L-type Ca^2+^ current density was uniform along the t-tubule and six times higher than in external membrane. In (H) and (K) the L-type Ca^2+^ current density was homogeneous throughout the cell surface. In (F–G) Na^+^/Ca^2+^ flux density was three times higher in the t-tubule and Ca^2+^ leak homogeneously distributed. In (H) Na^+^/Ca^2+^ exchanger and Ca^2+^ leak were homogeneously distributed via the sarcolemma. (L) Local Ca^2+^ time-courses with re-plot from experimental data [5]. The re-plots were taken along the scanned line at 0µm (*blue*), 3.96 µm (*green*) and 5.65 µm (*red*) from the near surface location. (M) Estimated SCH values with respect to the three flux distribution choices. In this numerical experiment the line-scan was positioned at 200nm away from the t-tubule membrane at the angle 120°. The scanned line in Cheng *et al.* experiment was located at 200nm from the surface of the t-tubule.

**Figure 5:**
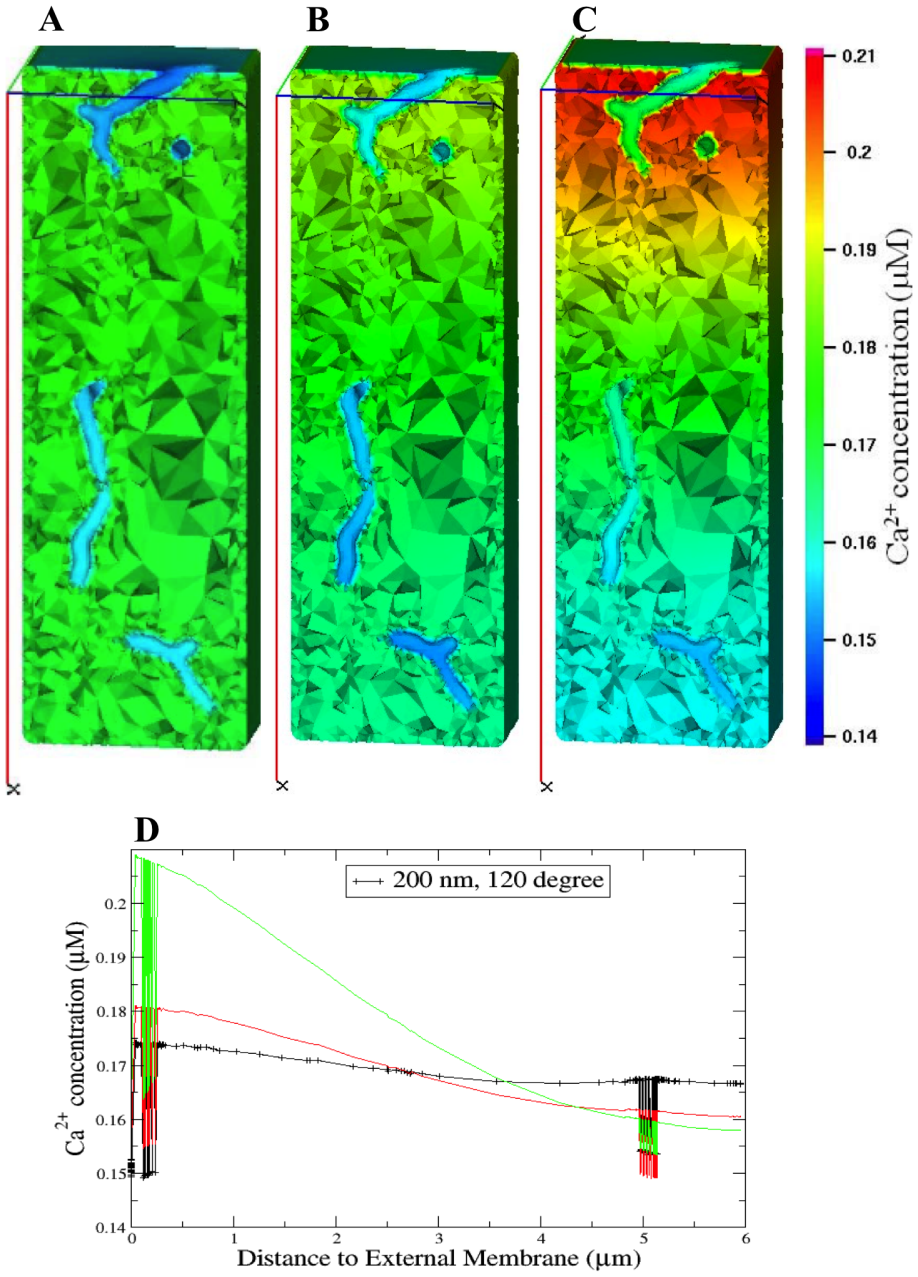
Calcium concentration distributions at Ca^2+^ peak in the presence of 100 µM Fluo-3. (A–C) 3-D views of the Ca^2+^ concentration distribution at Ca^2+^ peak of 76 ms. In (D) the spatial profiles at Ca^2+^ peak along the scanning line of interest are compared. L-type Ca^2+^ flux density heterogeneously distributed along the t-tubule membrane (A and *black plots* in D). L-type Ca^2+^ flux density six times higher and uniform via t-tubule membrane (B and *red plots* in D). L-type Ca^2+^ flux density uniformly distributed via the sarcolemma (C and *green plots* in D). In (A–B) Na^+^/Ca^2+^ flux density was three times higher in the t-tubule and Ca^2+^ leak homogeneously distributed. In (C) Na^+^/Ca^2+^ exchanger and Ca^2+^ leak were homogeneously distributed via the sarcolemma.

Model results in Figs. 4–5 were computed for conditions approximating those of the experiment by Cheng *et al.* [5], who examined Ca^2+^ signals in voltage-clamped rat myocytes in the presence of 100 µM Fluo-3 and pharmacological blockade of the SR (see Fig. 4L). The computed line-scan images and local Ca^2+^ time-courses are shown in Figs. 4F–4K.

These results demonstrate that with LCC heterogeneous or LCC six times higher in the t-tubule: (1) predicted Ca^2+^ concentration profiles were non-uniform when t<100 ms but the variations in [Ca^2+^]_i_ seem to be within the range of experimental noise in Fig. 4L; (2) [Ca^2+^]_i_ was more evenly distributed when t>100 ms, ( Figs. 4F–G, Figs. 4I–J, Video S1). To delineate further the suggested spatial differences in [Ca^2+^]_i_ (see Figs. 4F–H), we introduced a quantity called ‘spatial Ca^2+^ heterogeneity’ (SCH). The SCH is defined to be the difference of the maximal and minimal [Ca^2+^]_i_ values, normalized by the maximal value at given reference point along the line-scan (0.17 µm, 3.09 µm, 5.45 µm) in given moment t_j_, (see Figs. 4I–K). High SCH suggests non-uniform [Ca^2+^]_i_ distribution and SCH of zero indicates spatially uniform [Ca^2+^]_i_ distribution. The histogram in Fig. 4M shows that assuming LCC heterogeneous versus LCC 6 times higher in the t-tubule decreased SCH(t_Ica-peak_) by 1.6 folds, SCH(t_70 ms_) by 2.29 folds, SCH(_[Ca]i-peak_) by 2.34 folds, SCH(t_100ms_) by 2.87 folds, and SCH(t_200ms_) by 8.45 folds. These findings demonstrate that the predicted [Ca^2+^]_i_ distribution with LCC heterogeneous more closely resembles the Chang *et al.* experimental findings [5], (compare Figs. 4I and 4L). Finally, the model predicts strongly non-uniform Ca^2+^ transients when the LCC, NCX and Ca^2+^ leak fluxes were uniformly distributed throughout the cell surface (Figs. 4H, 4K, 4M). In addition, Video S1 (*see right panel*) demonstrates that here the Ca^2+^ signal spreads from the external membrane to the cell center as a continuum wave but after LCC channel closing (t∼72 ms) this wave faltered.

Predicted global [Ca^2+^]_i_ transient, Na^+^/Ca^2+^exchanger, and Ca^2+^ leak currents with LCC pathways heterogeneously distributed (as in Fig. 4F) are shown in Figs. 4E and 4C–D. Figure 4C demonstrates that: (1) the depolarization of cell membrane reversed the rest exchanger's direction (i.e. in the interval 0ms–70ms NCX operated in Ca^2+^ entry mode) while when repolarization occurred the flow of Ca^2+^ through NCX was reversed again (i.e. in the interval 70ms–400 ms NCX operated in Ca^2+^ exit mode); (2) upon returning to resting voltage of −50mV the exchanger's rate rapidly increased (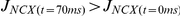) while 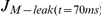 rate remained unchanged (note 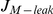 is not voltage-dependent) thus causing fast extrusion of Ca^2+^ out of the cell and subsequent sudden drop in the local and global [Ca^2+^]_i_. Figures 4F–K illustrate also that the global and all local Ca^2+^ transients reached the peak after ∼76 ms and that [Ca^2+^]_i_ levels were higher near the t-tubule mouth because the density of t-tubule branches was higher in this region and close topological proximity of the external membrane additionally increased the relative amount of the entering Ca^2+^. Due to the higher [Ca^2+^]_i_ gradient under the outer cell edge (t∼70ms) Ca^2+^ diffused toward the cell center and when 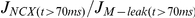 ratio along the cell membrane became approximately equal to 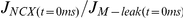 ratio a new equilibrium level of [Ca^2+^]_i_ (∼0.16 µM) was reached. Intracellular Ca^2+^ equilibrated faster when Ca^2+^ flux was more concentrated in the t-tubule membrane because [Ca^2+^]_i_ gradient near the t-tubule mouth was lower there than [Ca^2+^]_i_ gradient with Ca^2+^transporting complexes distributed homogeneously. Additional reasons for the observed rapid equilibrium of [Ca^2+^]_i_ may be that [Na^+^]_i_ was kept constant (in contrast to existing evidence for the presence of local sub-sarcolemmal [Na^+^]_i_ gradients on the action potential time-scale [33], [53]) or that the realistic distribution of NCX flux may be differ as assumed in the model. Finally, the results demonstrate that the computed average [Ca^2+^]_i_ peak of 160–185 nM (see Figs. 4E, 4I–4J), is comparable with the measured one of about 163 nM when the SR release and uptake were inhibited [5].

This model is also able to predict how the Ca^2+^ transients are regulated at different line-scan positions within this geometrically irregular micro-domain. Note, due to the technical limitations the Cheng *et al.* experiment is not able to suggest where exactly the scanned line is positioned with regard to the specific t-tubule, but the Cheng *et al.* measurements [5] strongly suggest the similarity of [Ca^2+^]_i_ at the peripheral and deeper cytoplasm when the SR activity is abolished. For this reason, we examined the Ca^2+^ profiles (LCC heterogeneous along the t-tubule, Fig. 4F) positioning the line-scan at 200 nm and angle 60° (see Fig. 3) or positioning the line-scan at 50, 100, 200, 300 or 400 nm at different angles. No visible differences in the visualized spatial Ca^2+^ profiles were found (*data not shown*).

The 3-D Ca^2+^ concentration distributions and spatial Ca^2+^ profiles at Ca^2+^ peak (76 ms) are shown in Figs. 5A–D. These model results demonstrate that the Ca^2+^ concentration near the external membrane decreased while [Ca^2+^]_i_ in the cell interior increased when Ca^2+^ transporters were uniformly distributed and after that heterogeneously redistributed. The jumps in Fig. 5D show the predicted local Ca^2+^ flux (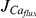) at [Ca^2+^]_i_ peak in the regions where the scanning line of interest is crossing the t-tubule membrane.

Additional interesting model findings are that: (1) large and steep [Ca^2+^]_i_ gradients were predicted inside the sub-sarcolemmal 3-D space (see Video S1); (2) the global Ca^2+^ time-course and time to [Ca^2+^]_i_ peak did not depend on whether Ca^2+^ fluxes are distributed homogeneously or heterogeneously along the sarcolemma (*data not shown*); (3) redistributing NCX flux uniformly via the sarcolemma was not able to alter significantly the predicted Ca^2+^ signals in Fig. 4F (*data not shown*).

In this study the value of 390 µm^2^ s^−1^ for diffusion coefficient of free Ca^2+^ and published buffer diffusion coefficients and parameters were used to compare the calculated Ca^2+^ signals with the Cheng's et al. fluorescence Ca^2+^ signals recorded in rats [5]. It has been suggest, however, that the effective diffusion of free Ca^2+^ in the cytosol (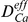) will be slowed down because the exogenous and endogenous Ca^2+^ buffers and free Ca^2+^ concentrations are able to affect Ca^2+^ diffusion strikingly [18], [19], [54]–[62]. The measurements of Allbritton et al. [54] report a value of 5–21 µm^2^ s^−1^ for 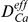 at low free 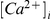 when a value of ∼223 µm^2^ s^−1^ is assumed for 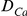. During Allbritton's et al. in vitro experiments Ca^2+^ sequestration by the SR, mitochondria and ATP was inhibited and only mobile calmodulin and stationary troponin C were present in the cytosolic extract. It is possible to estimate 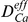 (0 µM Fluo-3, 0 µM ATP), using a simplified equation (see Eq. 13), because in this study the predicted maximal Ca^2+^ elevations were sufficiently small (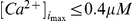) and the diffusion coefficients for Ca^2+^-bound and free mobile buffer forms were assumed equal [18], [19], [58], [59]. Our calculations predict a value of ∼8 µm^2^ s^−1^ for 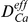 when 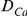 was 390 µm^2^ s^−1^ and 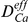 ∼6 µm^2^ s^−1^ when 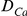 was 223 µm^2^ s^−1^. Therefore, the estimated value for 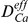 (∼8 µm^2^ s^−1^) is in reasonable agreement with the experimental observation.
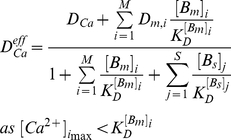
(13)


We could not find experimental data suggesting 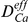 (260 µM ATP, 70 µM TN, 24 µM Cal) or 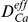 (100 µM Fluo-3, 260 µM ATP, 70 µM TN, 24 µM Cal) in the solution. Therefore, we used published concentrations of Ca^2+^ binding proteins and published diffusion and dissociation constants (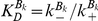) to estimate the effective diffusion constants of free Ca^2+^ in the presence of ATP or Fluo-3 and ATP in the cytosol. Our calculations indicate that adding 260 µM ATP in the solution accelerated 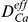 (∼10.4 µm^2^ s^−1^) and that 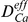 increased additionally when 100 µM Fluo-3 was added (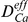 ∼66 µm^2^ s^−1^). Furthermore, our studies suggest that in the presence of 100 µM Fluo-3 and LCC heterogeneous Ca^2+^ binding and diffusion of ATP and calmodulin could not affected significantly the predicted [Ca^2+^]_i_ distributions (*data not shown*).

During simulations of SR Ca^2+^ release into the diadic cleft, a major effect of the stationary phospholipids Ca^2+^ binding sites has been suggested [17], [18]. To examine the impact of the phospholipids on the much smaller Ca^2+^ signals (arising from Ca^2+^ influx via Ca^2+^ current only), we included this buffer in our model. The results demonstrated that the phospholipids had only a limited effect on the calculated Ca^2+^ signals in the sub-sarcolemmal region (0 µM Fluo-3, 260 µM ATP, 24 µM calmodulin) and that this effect was even smaller when 100 µM Fluo-3 was included (*data not shown*).

### Model estimations of Ca^2+^ distribution under varying conditions

#### Ca^2+^ concentration changes in the absence of fluorescence dye and Ca^2+^ pathways heterogeneously distributed via the sarcolemma

**Figure 6:**
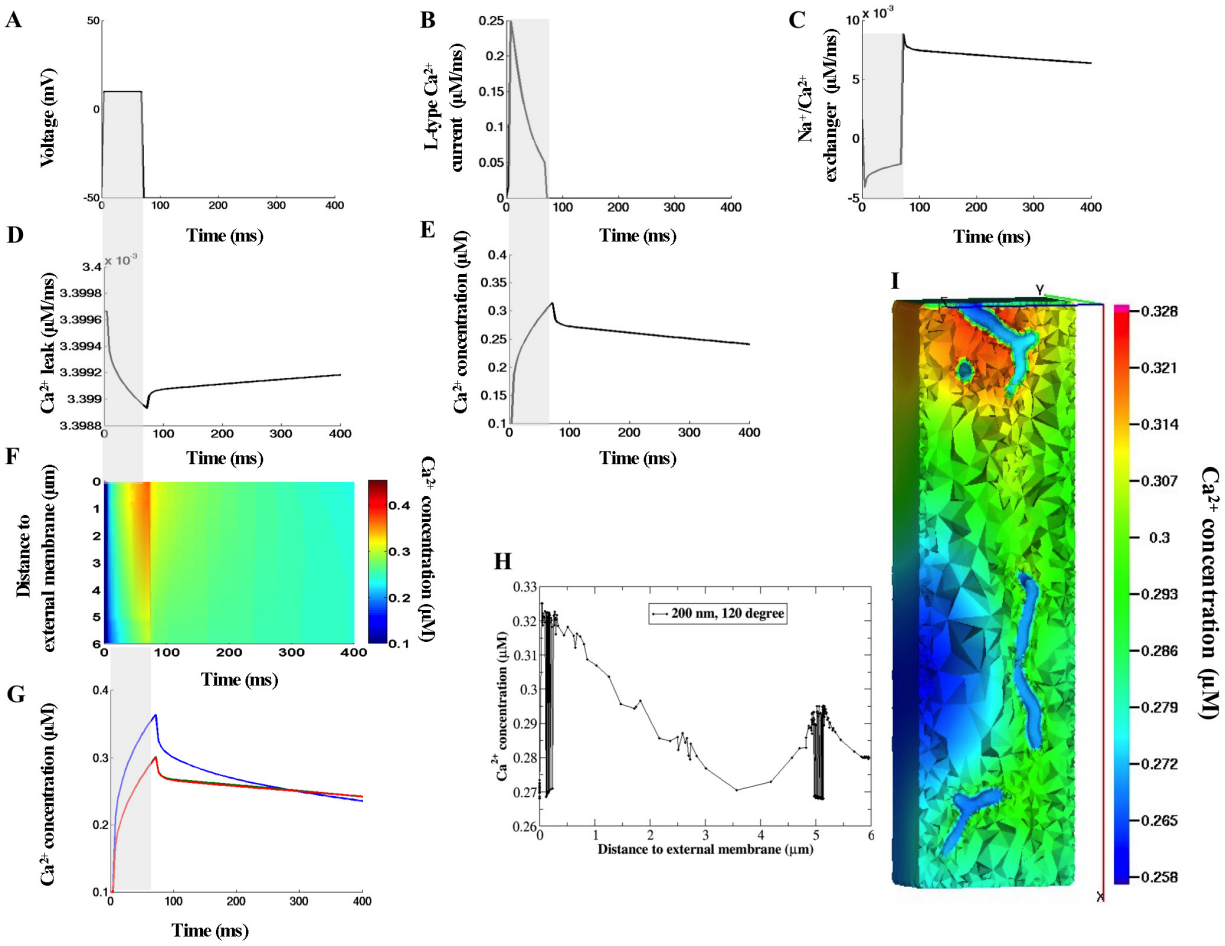
Model predictions in the absence of Fluo-3 and Ca^2+^ pathways heterogeneously distributed via the cell membrane. (A–B) The voltage-clamp protocol and whole-cell L-type Ca^2+^ current used in this set of simulations. (C–D) The predicted global Na^+^/Ca^2+^ and Ca^2+^ leak currents. (E–F) The global Ca^2+^ transient and Ca^2+^ concentrations visualized as line-scan image in the transverse cell direction. (G) Local Ca^2+^ transients taken at three featured spots along the scanning line (0.17 µm – *blue lines*, 3.09 µm – *green lines*, 5.45 µm – *red lines*). In (H–I) the spatial profile of Ca^2+^ along the scanning line and 3-D [Ca^2+^]_i_ distribution at Ca^2+^ peak of 76 ms are shown. In this numerical experiment the scanned line was positioned at 200nm away from the t-tubule membrane at the angle 120°.

**T**his model is also able to predict the spatial [Ca^2+^]_i_ signals that would occur in the absence of Fluo-3 (note experiments without fluorescent dye cannot be performed because of technical reasons). Since it has been suggested that the dye could not affect Ca^2+^ entry via L-type channels [63], [64], the same global LCC flux (as in Figs. 4–5) was used during this numerical experiment. Figures 6E–I show predicted Ca^2+^ signals arising from the ionic influx via L-type Ca^2+^ channels during voltage-clamp stimulation with LCC and NCX pathways heterogeneously distributed (as in Fig. 4F). The calculated global NCX and Ca^2+^ leak currents are shown in Figs. 6C–D, respectively.

The model predicts here that the spread and buffer capacity of 100 µM Fluo-3 were able to mask completely the pronounced spatial non-uniformities in [Ca^2+^]_i_ distribution that will occur during the Ca^2+^ influx when the SR Ca^2+^ metabolism is inhibited [5]. Figure 6 also illustrates, that detectible differences in [Ca^2+^]_i_ are found when t<150 ms and that [Ca^2+^]_i_ was more evenly distributed when t>150 ms, (see also Video S2, *left panel*). The removing of the mobile Ca^2+^ dye from the solution increased SCH(t_Ica-peak_) by 1.635 folds, SCH(t_70 ms_) by 2.63 folds, SCH(_[Ca]i-peak_) by 2.72 folds, SCH(t_100ms_) by 4.46 folds, and SCH(t_200ms_) by 8.65 folds (compare Fig. 6G with Fig. 4I where Fluo-3 was 100 µM). Additional findings are that: (1) upon depolarization NCX operated again in Ca^2+^ entry mode; (2) the reversal of current of NCX upon returning to resting voltage of −50mV increased additionally the current rate (*compare* Fig. 6C with Fig. 4C); (3) the global and local [Ca^2+^]_i_ peaks increased while the times to peak remained unchanged, i.e. ∼76ms (*compare* Figs. 6E and 6G with Figs. 4E and 4I); (4) the predicted 3-D [Ca^2+^]_i_ distribution at Ca^2+^ peak was strongly non-uniform (*compare* Fig. 6I and Fig. 5A); (5) upon repolarization initially [Ca^2+^]_i_ rapidly dropped, the following [Ca^2+^]_i_ decay was slow and the equilibrium was not reached even after 400 ms (Fig. 6E and Fig. 6G). Interesting model prediction is that larger, steeper and heterogeneous [Ca^2+^]_i_ gradients with regard to those predicted in the presence of 100 µM Fluo-3 could be expected in the narrow sub-sarcolemmal space (compare *left panel* in Video S1 with *left panel* in Video S2). Interestingly, no visible differences in the local Ca^2+^ time-courses were found again (as with 100 µM Fluo-3) when the line-scan was positioned 200 nm away from the t-tubule surface at angle 60° or at 50, 100, 200, 300 or 400 nm at different angles (*data not shown*).

#### Endogenous buffer mobility

**Figure 7:**
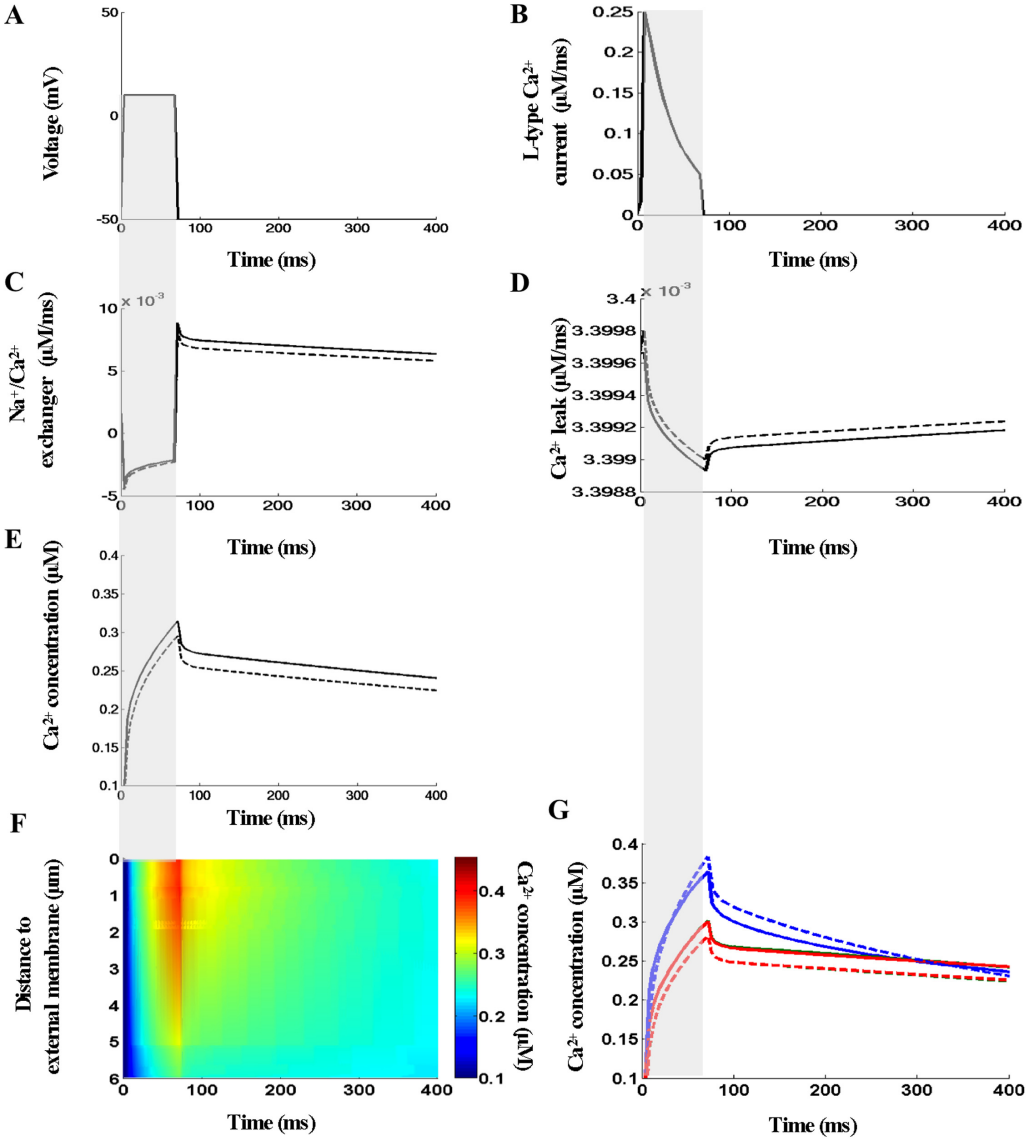
Effects of ATP and camodulin mobility on subcellular [Ca^2+^]_i_ signals. (A–B) The voltage-clamp protocol and whole-cell L-type Ca^2+^ current. (C–E) Quantitative comparison of the effects of buffer mobility on the global Na^+^/Ca^2+^ and Ca^2+^ leak currents and global Ca^2+^ transient (*solid lines* - CaATP and CaCal mobile, *dashed lines* - CaATP and CaCal stationary). (F) Line-scan image with CaATP and CaCal immobilized. (G) Quantitative comparison of the effects of buffer mobility on the local Ca^2+^ transients. During this numerical experiment Fluo-3 was zero, Ca^2+^ fluxes heterogeneously distributed via the sarcolemma, line-scan positioned at 200nm away from the t-tubule membrane at the angle 120°. Along the scanning line the featured spots were chosen to be the same as in Fig. 6G.

The conjecture made in the present model, that the endogenous calmodulin and ATP are mobile Ca^2+^ buffers, allowed us to examine how the mobility of these buffers would affect the Ca^2+^ dynamics and membrane flux time-courses within this irregular micro-domain. Figure 7 shows a simulation in which ATP (as CaATP) and calmodulin (as CaCal) were immobilized and Fluo-3 indicator removed from the solution. Under these conditions Ca^2+^ diffuses more slowly toward the center of the cell during the analyzed interval, resulting in a higher Ca^2+^ concentrations near the outer cell edge (*compare* line-scan images in Fig. 7F and Fig. 6F, *see* Video S2). Figure 7 also demonstrates that the peak of local Ca^2+^ transient near the external membrane increased while [Ca^2+^]_i_ peaks in the featured spots, 3.09 µm and 5.45 µm, decreased (see *dashed lines* in Fig. 7G). Here analysis suggests that assuming CaATP and CaCal stationary versus CaATP and CaCal mobile increased SCH(t_Ica-peak_) by 1.17 folds, SCH(t_70 ms_) by 1.47 folds, SCH(t_[Ca]i-peak_) by 1.49 folds, SCH(t_100ms_) by 1.92 folds, and SCH(t_200 ms_) by 3.54 folds (see Fig. 7G). The results also indicate that the predicted local changes in [Ca^2+^]_i_ gradients affected global NCX, Ca^2+^-leak and [Ca^2+^]_i_ time-courses: (1) upon repolarization global 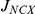 slightly decreased while 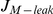 slightly increased; (2) [Ca^2+^]_i_ peak decreased but the time to peak remained unchanged (∼76 ms), (see *dashed lines* in Figs. 7C–E). Finally, these simulations demonstrated that under these conditions (i.e. immobilizing all endogenous Ca^2+^ buffers) Ca^2+^ could escape the tight control of membrane voltage in some sub-cellular regions giving rise of cytosolic Ca^2+^ wave initiated at the sarcolemma (see Video S2 *right panel* - near the outer cell edge Ca^2+^ wave was initiated (t∼60 ms) but very soon after ∼12ms this wave faltered).

#### Contribution of Na^+^-Ca^2+^ exchange to sub-cellular excitation-contraction coupling

**Figure 8:**
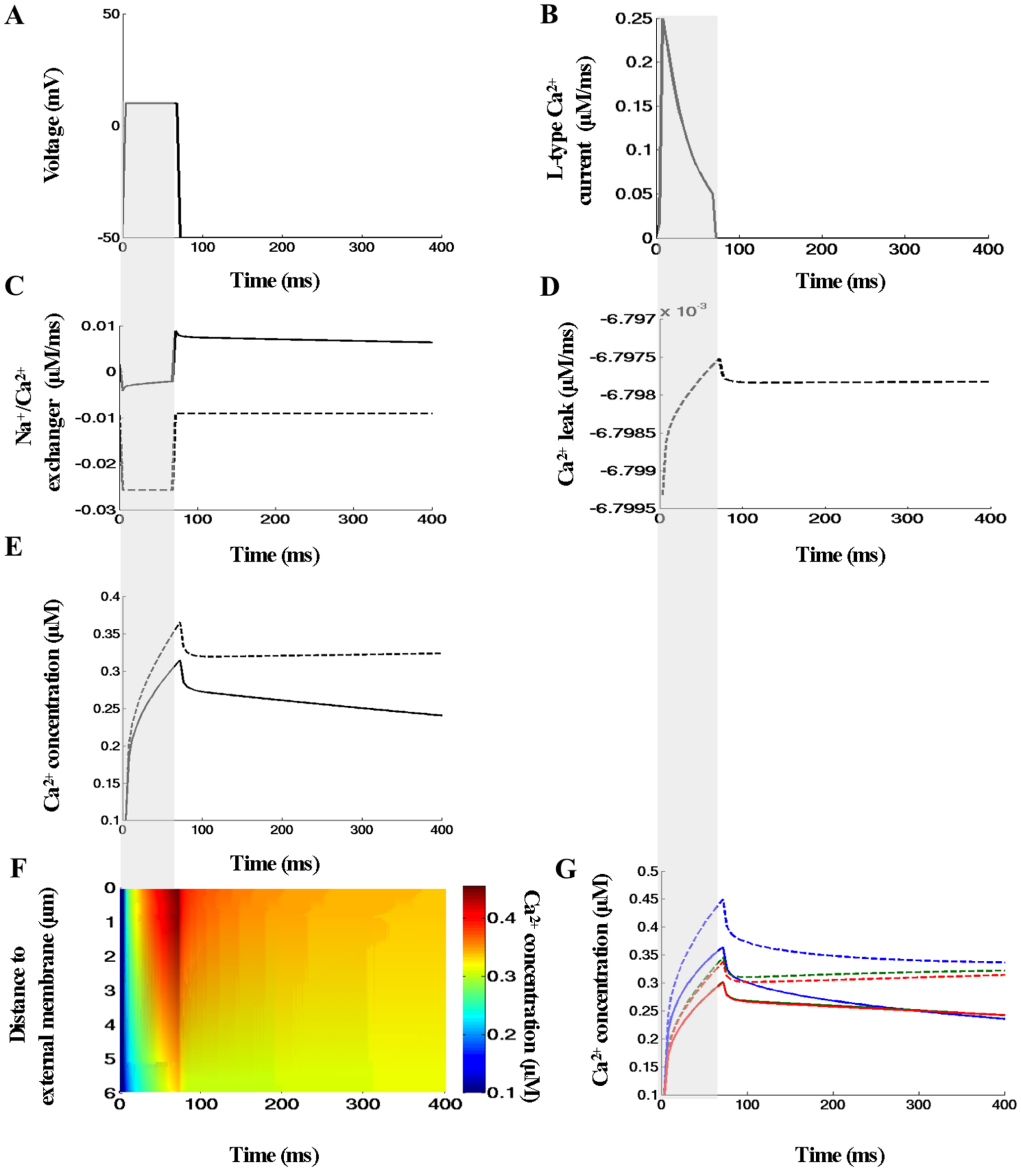
Effects of reduced extracellular [Na^+^] on subcellular [Ca^2+^]_i_ signals. (A–B) The voltage-clamp protocol and whole-cell L-type Ca^2+^ current. (C) Quantitative comparison of the effects of changes in [Na^+^]_e_ on the global Na^+^/Ca^2+^ flux (*solid lines* - [Na^+^]_e_ 140 mM, *dashed lines* – zero [Na^+^]_e_). (D) Predicted Ca^2+^ leak with zero [Na^+^]_e_. (E) Quantitative comparison of the effects of changes in [Na^+^]_e_ on the global Ca^2+^ transient. (F) Calcium concentrations visualized as line-scan images in transverse cell direction with zero [Na^+^]_e_. (G) Quantitative comparison of the effects of changes in [Na^+^]_e_ on local Ca^2+^ transients. In this numerical experiment Fluo-3 was zero, Ca^2+^ fluxes heterogeneously distributed via the sarcolemma, the line-scan positioned at 200nm away from the t-tubule membrane at the angle 120°. Along the scanning line the featured spots were chosen to be the same as in Fig. 6G.

In this study, we also examined the effects of NCX inhibition on the voltage-clamp induced Ca^2+^ signals in the absence of fluorescent indicator. The inhibition of NCX forward mode was achieved by removing extracellular sodium (i.e. 0 mM [Na^+^]_e_). To adjust Ca^2+^ flux via Ca^2+^ leak to match Ca^2+^ influx via NCX, so that at rest no net movement across the cell membrane to occur, we estimated Ca^2+^ leak constant (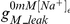) assuming [Na^+^]_e_ zero (see Table 3). Under these conditions NCX operated only in Ca^2+^ entry mode while membrane leak pumped Ca^2+^ out of the cell (see Figs. 8C–D *dashed lines*). Figures 8F–G (*see dashed lines*) show the predicted local [Ca^2+^]_i_ transients and the corresponding line-scan image. The line scan-image demonstrates that [Ca^2+^]_i_ distribution was again non-uniform but rather different compared to that predicted with 140 mM [Na^+^]_e_ (compare Fig. 8F with Fig. 6F). The results in Fig. 8G demonstrate that: (1) local [Ca^2+^]_i_ peaks in the featured spots (0.17 µm, 3.09 µm and 5.45 µm) increased and that this increase was more pronounced near t-tubule mouth; (2) upon repolarization [Ca^2+^]_i_ suddenly dropped because 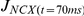 rate decreased while 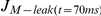 rate remained unchanged; (3) the decay of Ca^2+^ transient near the outer cell edge was slow; (4) [Ca^2+^]_i_ in the featured spots, 3.09 µm and 5.45 µm, begun slowly to increase when t>70 ms because 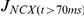 rate remained unchanged but 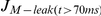 rate slightly decreased. With [Na^+^]_e_ zero versus 140 mM [Na^+^]_e_ SCHs increased: SCH(t_Ica-peak_) by 1.29 folds; SCH(t_70 ms_) by 1.4 folds; SCH(t_[Ca]i-peak_) by 1.43 folds; SCH(t_100ms_) by 1.65 folds; and SCH(t_200ms_) by 3.04 folds (see Fig. 8G). Furthermore, the NCX inhibition had visible effect on the global [Ca^2+^]_i_ transient, i.e. global Ca^2+^ peak increased and during membrane repolarization initially [Ca^2+^]_i_ suddenly decreased and after that rapidly equilibrated. In agreement with experimental data reported previously [65], the model also predicts increase in global [Ca^2+^]_i_ peak and no changes in the time to peak when [Na^+^]_e_ is completely substituted with 140 mM Li^+^, (see *dashed line* in Fig. 8E). New finding is that, with zero extracellular Na^+^, the Ca^2+^ signal spreads from the external membrane to the cell center as continuum Ca^2+^ wave initiated after 56 ms but very soon this wave faltered (see Video S3, *right panel*).

## Discussion

### The model with realistic transverse-axial t-tubule microanatomy provides new insights on how the Ca^2+^ signaling is regulated in rats

The current study attacks a difficult problem on how to incorporate the structural-based biological information, critical for the subcellular and cellular function, into sophisticated computational investigations. Pursuing this goal we developed a 3-D continuum model of Ca^2+^ signaling in rat ventricular cells that incorporates the realistic transverse-axial t-tubule topology and considers geometric irregularities and inhomogeneities in the distribution of ion-transporting proteins. The t-tubule micro-architecture was extracted from the Hayashi *et al.* two-photon imaging data in mice [6]. Because currently high-fidelity geometric models representing the realistic t-tubule micro-architecture in rats are not available, in this study we used the Yu's *et al.* geometric model in mice [28], [29]. On the basis of experimental data in rats the aqueous sub-cellular volume, accessible to Ca^2+^, was estimated to be ∼35–37% [33]. Since the Ca^2+^ signaling in cells is largely governed by Ca^2+^ diffusion and binding to mobile and stationary Ca^2+^ buffers [17]–[21], [23], [54]–[62], [66], [67], the effect of four Ca^2+^ buffers (Fluo-3, ATP, calmodulin, TN) was considered.

The model was validated against published experimental data on Ca^2+^ influx, membrane protein distributions and Ca^2+^ diffusion in rat cells treated with ryanodine and thapsigargin to inhibit the SR Ca^2+^ metabolism [2], [5], [10], [12], [15], [26], [35]–[43]. We found that with 100 µM Fluo-3 the results more closely resemble the Cheng's *et al.* experimental data [5] when the LCC density increases ∼1.7 fold along the t-tubule length and the NCX density is assumed three times higher in the t-tubule. An interesting finding is that with LCC six times and NCX three times higher and uniform in the t-tubule, the predicted fluctuations in the [Ca^2+^]_i_ profiles were within the range of experimental noise [5]. Strongly non-uniform spatial Ca^2+^ gradients and propagation of Ca^2+^ wave are predicted, not observed in Cheng *et al.* experiment, when the LCC and NCX were uniformly distributed along the sarcolemma.

The model studies with 100 µM Fluo-3 indicate also that the [Ca^2+^]_i_ gradients depend on the diffusion distances in the axial and cell surface directions. Thus, when the LCC were distributed uniformly the local Ca^2+^ peak in radial depth (5.96 µm) decreased from ∼1.5 fold while in the other cell directions (1 µm×1 µm) no significant changes were found. Redistributing the amount of Ca^2+^ pumped via the cell membrane (i.e. increasing LCC current density along the t-tubule) while keeping total Ca^2+^ flux unchanged, lowered Ca^2+^ gradients near the surface membrane and increased Ca^2+^ levels in the cell interior (see Video S1). The results also showed that with 100 µM Fluo-3 and Ca^2+^ flux heterogeneously distributed along the sarcolemma, the computed average [Ca^2+^]_i_ peak (160–185 nM) is comparable to the measured of about 163 nM [5] and that the NCX redistribution alone yields to qualitatively similar [Ca^2+^]_i_ profiles.

It should be noted, that in our previous work we used the simplified t-tubule geometry (assuming cylindrical shape) to simulate the Ca^2+^ dynamics in rats [19]. This idealistic t-tubule model also predicts the lack of systematic differences in the fluorescence Ca^2+^ signal when the Ca^2+^ transporters were distributed heterogeneously along the sarcolemma. Thus, the following question arises: How these new computational studies based on more realistic t-tubule structural model will further advance our current knowledge on the cell excitability and Ca^2+^ cycling in rats? First, in agreement with experiment [2]–[12] current study confirms that due to the branched t-tubule microstructure high and steep sub-sarcolemmal [Ca^2+^]_i_ gradients could occur throughout the whole cell volume [2]–[6], [33], (see Video S1). Note, Lu *et al.* idealistic t-tubule model predicts high and steep sub-sarcolemmal [Ca^2+^]_i_ gradients only in the transverse cell direction [19]. Second, our realistic t-tubule model predicts non-uniformities in [Ca^2+^]_i_ distribution along the depth of the t-tubule when t<100 ms (see Fig. 4F and Video S1 *left panel*) while this was not the case when the t-tubule geometry is assumed cylindrical (Fig. 4g in Lu *et al.*, [19]). Third, interesting finding is that no visible differences in the local Ca^2+^ profiles are predicted when the line-scan was positioned at different. Note, due to the technical limitations the Cheng *et al.* experiment is not able to suggest where and how exactly the scanned line is positioned with regard to the specific t-tubule [5].

A surprising and important finding of this study is that the spread and buffer capacity of 100 µM Fluo-3 were able to mask completely the pronounced spatial [Ca^2+^]_i_ non-uniformities that would occur during the Ca^2+^ influx in the absence of dye (see Video S2 *left panel* - SR Ca^2+^ metabolism inhibited, LCC and NCX transporters heterogeneously distributed). Here the simulations demonstrated that with zero Fluo-3 the local and global Ca^2+^ peaks increased while the time of Ca^2+^ rise remained unchanged. The predicted sub-sarcolemmal [Ca^2+^]_i_ gradients were heterogeneous along the cell membrane and larger and steeper compared to those with 100 µM Fluo-3. The NCX and Ca^2+^ leak time-courses were also affected due to increased local free [Ca^2+^]_i_ levels. It is interesting that under these conditions no differences in the local Ca^2+^ time-courses were found (as with 100 µM Fluo-3) when the line-scan was positioned at different angles and distances. To test further the model we also examined how the mobility of endogenous Ca^2+^ buffers (ATP and calmodulin) and altered extracellular Na^+^ ([Na^+^]_e_) would affect the Ca^2+^ signals in the absence of fluorescence dye when the Ca^2+^-transportes are heterogeneously distributed. The results showed that when ATP and calmodulin were immobilized Ca^2+^ diffuses slowly toward the center of the cell, resulting in higher Ca^2+^ concentrations near the outer cell edge. When the NCX forward mode was inhibited (assuming [Na^+^]_e_ = 0 mM) the local [Ca^2+^]_i_ peaks increased and this increase was more pronounced near the outer cell edge. New findings are that under these conditions near the outer cell edge Ca^2+^ wave was initiated while this was not the case when ATP and calmodulin were mobile and [Na^+^]_e_ 140 mM (see Video S2 and Video S3).

Taken together, these studies provide compelling evidence that (1) the exogenous Fluo-3 acts as a significant buffer and carrier for Ca^2+^, and that (2) the use of 100 µM Fluo-3 during the experiment can sensitively alter the realistic Ca^2+^ distribution. A new the question, however, arises: Based on the above model findings what could be the underlying mechanism(s) for the predicted heterogeneous Ca^2+^ concentrations gradients in the absence of Fluo-3? A reasonable answer is that the Ca^2+^ movement and distribution inside the cell rely strongly not only on the specific cell micro-architecture and Ca^2+^ transporters distribution but also on the presence of endogenous mobile and stationary Ca^2+^ buffers (ATP, calmodulin, troponin C - known to affect strikingly the Ca^2+^ dynamics) [12], [17]–[21], [23], [27], [67]. In support of this hypothesis, our simulations studies revealed that in the absence of Fluo-3: (1) the stationary Ca^2+^ buffer troponin C imposed stronger diffusion barrier for Ca^2+^ that resulted in larger and steeper sub-sarcolemmal Ca^2+^ gradients; (2) in the cell interior, owing on their sheer buffering capacity, Ca^2+^ buffers (troponin C, ATP, calmodulin) tended to slow down additionally the propagation of Ca^2+^ so that ATP and calmodulin spreading alone was not able to contribute the spatially uniform Ca^2+^ profiles to be achieved; (3) immobilizing the endogenous Ca^2+^ buffers slowed down the Ca^2+^ movement from the cell periphery to the center leading to build-up of large sub-sarcolemmal Ca^2+^ gradients and subsequent initiation of Ca^2+^ wave. It is important to mention that the Lu *et al.* idealistic t-tubule model predicts completely different 3-D [Ca^2+^]_i_ distributions with zero Fluo-3, SR Ca^2+^ metabolism inhibited and Ca^2+^ transporters heterogeneously distributed [19].

### Limitations of the model and future directions

Important limitations of the current modeling approach are: (1) the relatively small size of the model compartment that contains only a single realistic t-tubule shape and spans by just a half-sarcomere inside the ventricular myocyte; and (2) the assumption that the model compartment is a repeating unit inside the cell. The structural studies, however, provide evidence that in rodent ventricular myocytes the realistic t-tubule network is quite complex, (see Fig. 1A), [6]. The above limitations can be overcome in the future by extending the current geometric model toward more realistic models containing several t-tubules, whole-cell t-tubule network or other sub-cellular organelles (including mitochondria, SR, nuclei). This would allow building an improved geometric models representing more correctly the cell segment of interest and help to gain further insights of how the Ca^2+^-signaling in rat ventricular myocytes is regulated in the absence or presence of SR Ca^2+^ release and uptake [20]–[23], [66], [67]. However, it is equally important to mention here, that although the limitations (1–2) this model in a first approximation may yield insights across the whole-cell scale of biological organization. It allows simulating the global Ca^2+^ signal (computed from the line-scan image in Fig. 4F) that roughly would reproduce the whole-cell Ca^2+^ transient in the Cheng *et al.* experiment due to observed spatial similarities in [Ca^2+^]_i_ (see Fig. 1B–1C in [5]). This assumption enables also investigating how the whole-cell Ca^2+^ signal is regulated by the realistic t-tubule microanatomy, by 3-D distributions of ion-transporting proteins, by mobile dye or endogenous mobile and stationary Ca^2+^ buffers. It should be noted, that the common pool modeling approaches could not be used to investigate these effects [10], [24], [25].

Important limitation of this study is also that we assume that the ion flux pathways are continuously distributed throughout the t-tubule membrane. Immunohistochemical studies, however, suggest that L-type Ca^2+^ channels appear to be concentrated as discrete clusters in the dyadic clefts (narrow spaces between LCC and RyR) distributed regularly along the t-tubule membrane at relatively small distances of ∼0.68 µm, [12], [38], [68]. It is interesting to mention that in contrast to Soeller and collaborators data in rats [12], Hayashi *et al.* data in mice [6] suggest that the dyadic clefts are distributed irregularly along the t-tubule branches. In addition, NCX appears to be absent from the longitudinal tubules [42]. Thus, the above data clearly imply that localized concentration of LCC or NCX flux pathways could result in larger sub-sarcolemmal Ca^2+^ gradients and local membrane currents that will affect differently the spatial Ca^2+^ profiles as predicted with the current model. Further extending of our current t-tubule model toward more realistic geometric models containing dyadic cleft topology and L-type Ca ^2+^ channel clustering could help to better understand how the Ca^2+^ signaling is regulated in the heart.

Finally, in the present model the effects of two endogenous Ca^2+^ mobile buffers (ATP, calmodulin) and one stationary Ca^2+^ buffer (troponin C) were considered only. The ventricular cells, however, contain other stationary Ca^2+^ buffers (including phospholipids, myosin, calsequestrin) or small and high mobile Ca^2+^ binding molecules (ADP, AMP) that were not included in the model (or may be other stationary and mobile buffers that have not been identified yet), [18], [24], [69].

### Conclusions

Simulations presented in this study demonstrate that the more accurate knowledge of transverse-axial t-tubule microanatomy and protein distributions along the sarcolemma is important to better understand the mechanisms regulating the excitation-contraction coupling in rat ventricular myocytes. The results demonstrate that Ca^2+^ movement from the cell membrane to the cell interior relies also strongly on the presence of mobile and stationary Ca^2+^ buffers, including the Ca^2+^ indicator dye. The key findings are: (1) the model predicts lack of detectible differences in the fluorescence Ca^2+^ signals at the peripheral and deep myoplams when the membrane Ca^2+^ flux is heterogeneously distributed along the sarcolemma; (2) 100 µM mobile Fluo-3 is able to mask the pronounced spatial non-uniformities in the [Ca^2+^]_i_ distribution that would occur when the SR Ca^2+^ metabolism is inhibited; (3) during the Ca^2+^ influx alone, large and steep Ca^2+^ gradients are predicted in the narrow sub-sarcolemmal space (∼40–50 nm in depth); (4) in rodents the branched t-tubule topology, the punctuate spatial distribution of Ca^2+^ flux along the sarcolemma and the endogenous Ca^2+^ buffers actually function to inhibit Ca^2+^ waves. Improved functional and structural computational models are needed to guide the experiments and to test further our understanding of how the t-tubule microanatomy and protein distributions regulate the normal cell function or cell cycle under certain pathologies. To our best knowledge, this study is the first attempt to use the finite element methods to investigate the intracellular Ca^2+^ responses in physiologically realistic transverse-axial t-tubule geometry.

## Supporting Information

Video S1 100 µM Fluo-3: Calcium flux heterogeneously distributed (*left panel*); Calcium flux higher and uniform in the t-tubule (*middle panel*); Calcium flux uniformly distributed (*right panel*).
(5.45 MB AVI)

Video S2 Zero Fluo-3 and calcium flux heterogeneously distributed: ATP and calmodulin mobile (*left panel*); ATP and calmodulin immobile (*right panel*).
(4.05 MB AVI)

Video S3 Zero Fluo-3 and calcium flux heterogeneously distributed: Extracellular sodium 140 mM (*left panel*); Zero extracellular sodium (*right panel*.)
(4.07 MB AVI)
